# Beyond Valence and Arousal: Distributed Facial Patterns Linked to Specific Emotions Cannot be Reduced to Core Affect

**DOI:** 10.1007/s42761-025-00311-2

**Published:** 2025-06-20

**Authors:** Rotem Simhon, Nachshon Meiran, Shaul Shvimmer, Liron Amihai, Yitzhak Yitzhaky, Jonathan Rosenblatt, Michael Gilead

**Affiliations:** 1 School of Psychology, Tel Aviv University, Tel Aviv, Israel; 2 Department of Psychology, Ben Gurion University of the Negev, Be’er Sheva, Israel; 3 Electro-Optics Engineering Department, School of Electrical and Computer Engineering, Ben Gurion University of the Negev, Be’er Sheva, Israel; 4 Department of Industrial Engineering and Management, Ben Gurion University of the Negev, Be’er Sheva, Israel; 5 Department of Psychology, Ben Gurion University of the Negev, Be’er Sheva, Israel

**Keywords:** Discrete emotions, Core affect, Facial physiology, Facial patterns, Emotional state

## Abstract

Emotions are central to human life and, as such, are of primary interest in basic psychological research. There is widespread agreement that emotions involve subjective experiences that can be described with discrete natural language labels and often involve changes in bodily states, but there is ongoing debate about how specific and differentiated these bodily states are, and how they relate to emotional labels. Recent work showed that objective measures derived from the face can be used to accurately classify discrete emotional labels. However, it remains possible that the facial patterns associated with discrete emotions merely convey valence and arousal (i.e., “core affect”) and that this information can be utilized to deduce discrete emotion categories. In light of this, in the current work, we examined whether the facial patterns that reliably distinguish between emotional states are reducible to valence and arousal. Our findings support the position that the human face contains rich information that can be used to predict people’s emotional states and that this information is not reducible to core affect. We discuss the implications of this work to the debate concerning the nature of emotions.

Emotions are central to human life and, as such, are of primary interest in psychological research. It is widely held that emotions often involve subjective experiences that can often be expressed using discrete natural language labels (henceforth, simply “conscious emotional experience”; e.g., “I am *afraid*”). Another common belief is that emotions are often coupled with changes in bodily states such as motor action (e.g., approach or avoidance tendencies), facial responses (e.g., crying), and autonomic activity (e.g., heart rate changes; Ekman & Davidson, 1994; Mulligan & Scherer, 2012; Panksepp, 2004). However, the question of how specific and differentiated these bodily states are—and how they relate to discrete emotional labels—has evaded scientific consensus for more than a century.

William James (1884) suggested that subjective experiences in emotions are marked by “distinct bodily expressions.” Likewise, Lange (1885) suggested that emotional stimuli evoke specific physiological reactions, and the perception of those changes constructs the emotional experience. However, the idea that there are distinct bodily states associated with distinct subjective states has not been without its critics. Canon (1927) argued that feelings and bodily responses are independent components of emotions and that emotions have unitary and highly similar autonomic responses. Similarly, Schachter and Singer (1962) argued that the same bodily state can produce dramatically divergent emotional states, depending on one’s cognitive interpretation of the events in which the emotional reactions arise. Since then, numerous findings and theories were offered in support of the distinct bodily state’s view (Ekman et al., 1983; Fehr & Stern, 1970; Lench et al., 2011; Nummenmaa et al., 2014; Stemmler, 2004) or to the contrary (Barret, 2006; Cacioppo et al., 2000; Mauss & Robinson, 2009; Taylor, 1991).

From the historic debate on the relationship between emotion and body, one thing is clear: There are bodily states that often accompany our conscious emotional experiences. However, the views concerning the specific relation between the two phenomena markedly differ:

(i) The most minimal theory argues that knowing something about a person’s bodily state (e.g., autonomic arousal) only provides information about whether one’s conscious experience is emotionally arousing or not (e.g., Shachter & Singer, 1962).
(ii) A slightly less minimal theory is that some bodily states (e.g., overt facial expressions) convey some basic information about the valence (i.e., pleasantness) and arousal of the conscious emotional state. This view can be termed the “core affect” perspective (e.g., Barret et al., 2019).
(iii) The maximalist account will suggest that specific bodily states can be used to accurately predict the specific, discrete emotional label by which a person will describe their emotional experience (see Ekman et al., 1983 for a theory that is consistent with the maximalist view).

This classic debate between minimalist and maximalist accounts remains at the heart of emotion science (Barret, 2006; Ekman, 2016; Friedman, 2010; Kreibig, 2010). Empirical attempts to support (or refute) the maximalist view have focused on examining various types of bodily responses, such as autonomic arousal (Christie & Friedman, 2004; Greenwald et al., 1989) and vocal bursts (Cowen et al., 2019). However, arguably, the most central front in these “emotion wars” is the battlefield that is the human face.

This view that specific facial patterns are associated with specific conscious emotional states (the maximalist account) dates back to Darwin (1872), who claimed that some emotions have universal facial expressions and proposed principles explaining why particular expressions occur for particular emotions. Darwin further believed that such expressions are overt and serve to communicate one’s emotions to others. This claim was popularized by Ekman (1984) who provided evidence for universality in facial overt expressions of several “basic emotions” (e.g., fear, disgust). Ekman and Friesen (1971) conducted research on indigenous populations with minimal exposure to Western culture and used choice-from-array tasks to show that both indigenous and Western participants typically select the same label for a given emotional expression. Since then, there have been numerous attempts to map specific overt facial expressions into discrete emotional labels (Cowen & Keltner, 2020; Ekman, 1993; Eckman, 1997; Liu et al., 2022).

However, the maximalist account has been forcefully challenged by a recent cross-cultural investigation of emotional labeling in indigenous populations. In these recent investigations, it was shown that when participants are asked to provide labels of overt facial expressions in an open-ended manner (rather than perform a choice-from-array task), there is simply no cross-cultural agreement concerning the appropriate emotional label for a given expression. The only robust cross-cultural agreement pertains to the valence and arousal of the emotional expression (Crivelli et al., 2016; Crivelli et al., 2017; Gendron et al., 2014; Gendron et al., 2018)—and this minimal information can often be used to “correctly” infer the specific emotion label in a forced-choice context. In other words, this line of work provided compelling evidence for the (relatively minimalist) “core affect” perspective.

These findings imply that humans are (perhaps surprisingly) limited in their ability to collect rich information from facial expressions beyond information concerning core affect. Importantly, while most of the research in these areas has examined the question of perceptible, overt facial expressions, the question of the relationship between emotional and bodily (in this case, facial) states need not be limited to overt expressions easily detected by the naked eye.

It remains possible that more sensitive detectors than human vision (i.e., sensitive cameras) and stronger inferential methods (e.g., machine learning algorithms) can be used to accurately detect discrete emotional labels from the face, by detecting both overt, perceptible, and covert, non-perceptible information—thus showing that there is information in the human face that is related to discrete subjective emotion labels.

## The Current Research

In a recent study by Shvimmer et al. (2022), we developed a new technological solution for classifying whether individuals watched neutral, disgust, joy, fear, or sexual-arousal eliciting videos. This method applied machine learning to physiological features gleaned from optical imaging of participants’ faces as they watch emotion-eliciting videos. It benefits from the different wavelength sensitivity of three different cameras: RGB (regular), active near-infrared (NIR), and long-wave infrared (thermal). Using this approach, we record (both perceptible and non-perceptible) physiological changes across the human face to classify one’s emotional states. These physiological changes include blood flow, hemoglobin concentration, temperature, and possible spontaneous facial expressions. Thus, it captures different kinds of physiological facial changes. The five-class classification results of this method showed an identification rate of 43.62% on average (vs. 20% chance level).

The fact that objective measures derived from the face can accurately classify discrete emotional labels that represent participants’ subjective emotional states seems to be consistent with the idea that different emotions have different biological bases; as such, one might see these findings as being more consistent with the view that that specific bodily states can be used to accurately predict the specific, discrete emotional label by which a person will describe their emotional experience.

However, since there is growing evidence that what is visible in (overt) facial expressions is merely valence and arousal (Crivelli et al., 2017; Gendron et al., 2014; Russell, 2003), it is quite possible that the patterns captured by our classifier simply reflect some linear or non-linear combination of valence and arousal (Russell, 1995; Russel, 2003). In other words, like humans, the classifier may be able to attain accurate classification on the forced-choice classification task by relying on the “core affect” information that is already known to be present in the face.

Thus, if valence and arousal can explain the emotion classification results, this will constitute further support to the *core affect view*, which represents a relatively minimalistic and unitary approach to emotions*.* If, however, valence and arousal do *not* suffice to explain the classification results, this will be more in line with the picture painted by the maximalist view, which, as noted, suggests that specific bodily states can be used to accurately predict the specific emotional label by which a person will describe their emotional experience.

## Method

Our novel solution for classifying discrete emotion labels via transdermal cardiovascular spatiotemporal facial features is further detailed in a methodological paper (Shvimmer et al., 2022). We briefly describe it herein.

### Apparatus

In order to record participants’ faces, we used three cameras, with different wavelength sensitivities: (i) RGB camera, Sony Alpha 6000; (ii) Near-Infra-Red (NIR) camera, ELP 2 MP; and (iii) Thermal Imaging Long-Wave Infra-Red (LWIR) camera, OPTRIS PI450 camera. Participant’s heads were mounted on a chin rest (Good-Light Head and Chin Rest Double Screw Clamp) to reduce movements as much as possible. The experiment was conducted on a PC with an Intel i7-9700 processor with 32 GB RAM. It was performed using custom-written software in MATLAB.

### Stimuli

We curated a database containing 150 videos that portrayed a wide range of psychologically meaningful events. These included videos featuring spiders and snakes, adorable animals, art and architecture, the marvels of nature, natural disasters, explosions and warfare, feces and vomit, sexual activities, accidents and close calls, and various other subjects. The database contained 30 of each of the following categories: Disgust, Fear, Sexual Desire, Amusement, and Neutral. The categories disgust, fear, and amusement were taken from Cowen and Keltner (2017) database. We chose 30 videos from each category by the following criteria: the videos with the higher percentage of concurrence (eliciting the same category of emotion), the smallest overlap with other terms in the semantic space (i.e., videos that were rated as least eliciting other), and in the range of 4–15 s. Neutral videos were taken from Samson et al. (2016) and were chosen by the same criteria.

During our search for a suitable database, we did not find a database for the sexual desire category that fitted our criteria. Therefore, we performed an online study to gather 30 video clips that elicit sexual desire. We ran an online study using 44 participants recruited through Amazon Mechanical Turk (MTurk) who received a compensation of $2.5. Three participants were excluded based on failure to comply with task demands. The final analysis included 41 participants (14 females; 27 males). Participants ranged in age from 22 to 66 (*M* = 39.07; *SD* = 9.83). Overall, 40 videos were gathered from the Pornhub website (https://www.pornhub.com), and each of the participants watched all 40 videos. All of the participants indicated which category they felt while watching each of our 40 videos (they were able to choose more than one). In addition, the participants rated each video on two dimensions, valence, and arousal, on a scale of 1 to 9. We chose 30 video clips by the following criteria: the videos with the higher percentage of concurrence (eliciting the sexual desire emotion), the smallest overlap with other terms in the semantic space, and in the range of 4–15 s.

The resulting video set was composed of five emotional categories and contained clips that lasted on average 7.5 s: Disgust (*M* = 7.13;* SD* = 2.64), Fear (*M* = 7.80;* SD* = 3.59), Amusement (*M* = 8.13;* SD* = 3.04), Neutral (*M* = 7.46;* SD* = 2.54), and Sexual Desire (*M* = 7.66;* SD* = 2.50). In order to eliminate a classification based on the difference in the length of the videos, we tested all possible sets of two emotions using *t*-tests to check for duration differences. No significant difference was found despite the resultant alpha inflation associated with multiple *t*-tests.

### Participants

Given that there is currently no established methodology for conducting power analysis for machine learning analyses, and considering that our study pertained to a novel imaging modality with limited prior research, we attempted to acquire the largest possible sample size given practical constraints (i.e., a time-period of one year).

A total of 110 Israeli undergraduate students at Ben-Gurion University (63 females, mean age = 24.6) participated in the experiment for course credit or money (50 NIS). Of the 110 participants included in the study, 93% identified as heterosexual, 4% as bisexual, 2% as gay or lesbian, and 1% refused to answer. The research was approved by the Faculty Ethics Committee. All participants declared being native speakers of Hebrew and having normal or corrected to normal vision without glasses (since their faces were recorded and glasses cover parts of the face). We assured that participants understood the instructions properly by asking each participant during the debriefing to explain in his or her words the instructions of the experiment.

### Procedure

Participants viewed 150 emotion-eliciting videos in a pseudo-random order. The videos were divided into blocks, and each block contained five videos of the same emotional category. Overall, 30 blocks were generated, five of each emotion. Four different block sequences were created; in each sequence, the order of the blocks and the videos inside each block were in a pseudo-random order. The experiment was programmed in MATLAB using custom-written software that was developed to run the experiment. Following each video, participants were asked three questions: (i) what is the most dominant emotion you experienced while watching the last video (Disgust, Fear, Neutral, Sexual Desire, Amusement, and None of the following emotions); (ii) to what extent does this make you feel stimulated. The scale ranged from 1 (*more subdued)* to 9 (*more stimulated)*; (iii) to what extent does this make you feel pleasant. The scale ranged from 1 (*very unpleasant)* to 9 (*very pleasant)*; the rating for both of our affective dimensions was obtained on a nine-point Likert scale with the number 5 anchored at neutral.

The experimenter explained the instructions to the participants and stayed in the room during a short training phase (a single neutral video that was excluded from the database). The experimenter ensured the participant understood the task and then left the room. While watching the videos, participants’ faces were recorded using three cameras with multiple wavelengths: RGB (regular), active near-infrared (NIR), and long-wave infrared (thermal). After finishing the experiment, participants were shortly debriefed and released.

Participants knew that they were part of an experiment involving thermal imaging of the face with regards to emotions. They did not assume they needed to produce facial movements, their task was to report which emotion they felt and how bad vs. good the videos made them feel using the keyboard keys. They were not told to produce facial movements and were told that while they are being recorded with a set of unique cameras, such as cameras that detect heat, the only people who will have access to these videos are members of the research lab for research purposes.

### Data Processing

As a first stage, and in order to avoid length-induced bias in the classification learning process, the recorded face videos were sliced to include only their initial 120 frames, yielding consistent, same-size face video clips, each of a 4-s duration, to process. Overall, this process yielded a total of 130 face videos per experiment for each subject (20 videos where the were the emotion eliciting event did not occur within this initial 4-s window were excluded out of the original 150 recorded) for further analysis. Then, per each recorded video, accurate face regions in the RGB and NIR channels were located using the pre-trained machine learning-based Viola-Jones classifier, implemented by OpenCV (Viola & Jones, 2001; Viola et al., 2004). Afterward, spatial averaging was performed to improve temporal resolution in each pixel, followed by a heart frequency detection algorithm, peaks and troughs finding algorithm, and construction of transdermal spatialtemporal multi-spectral (TSTMS) features, engineered to maximize relevant physiological changes. For a more detailed explanation of the data processing, see Shvimmer et al. (2022).

### Classification Method

The spatialtemporal multispectral features were used as input for a classification algorithm. We performed five-class (i.e., Amusement (A), Disgust (D), Fear (F), Sexual Arousal (S), and Neutral (N)) multiclass classification using the extracted TSTMS features via the one-vs.-one (OvO) approach with the CatBoost machine learning classifier by Yandex (Dorogush et al., 2018). We relied on the Leave-One-Subject-Out Cross-Validation (LOOCV) approach since it uses only a single subject’s recorded face videos as test data in each iteration, while all other 109 subjects recorded face videos are used as training data.

### Data Structure

Our data consists of three segments. The first segment contains the self-reports of the affective dimensions per video, averaged across subjects. The second segment contains the self-reports of the discrete emotion labels per video, averaged across subjects. The third segment contains the classification output, i.e., the probability of a particular video being classified as evoking a particular emotion, averaged across subjects. Notably, the classification data is dependent since it comprised a probability matrix, but the probabilities of the five emotion categories sum up to 1.

### Transparency and Openness

We report how we determined our sample size, all data exclusions, all manipulations, and all measures in the study, and we follow JARS (Kazak, 2018). All data, analysis code, and research materials are available upon request. Data were analyzed using R, version 4.2.2. This study’s design and its analysis were not pre-registered.

## Results

### Manipulation Check

Before examining our main questions, we tested whether our videos reliably elicited self-reports of distinct emotional experiences for each of our five discrete emotional categories. To do so, we examined the accuracy rates for each of our five emotional categories. The accuracy rate is defined as the probability of a video being labeled according to the validated category. Each of our videos elicited a higher than chance (20%) accuracy rate, and the overall accuracy rate was 71.4%, specifically: Neutral 76.3%, Disgust 74.5%, Fear 63.9%, Amusement 78.0%, and Sexual Desire 64.0%. These results demonstrate that all five categories of emotional experience are reliably elicited by the videos. However, it is important to note that the accuracy for sexual desire, while above chance, is less robust compared to other emotions. This could result from differences between the samples in the validation pilot vs. the main study, or the possibility that the stimuli used did not elicit enough sexual arousal in participants. It is possible that other types of pornographic content could produce stronger emotional correlates. Moreover, participants were able to report that they felt “none of the above” if the video evoked an emotion that is not one of five options, and “neutral” if the video did not generate any sort of emotion in them. The scale ranged from 1 to 9, with the midpoint being 5. The mean valence of the Neutral videos was 5.21 (vs. 5.51 for sexual desire, 6.57 for the amusement videos, 3.73 for the fear videos, and 2.82 for the disgust videos). Thus, all in all, participants reported the neutral videos as being indeed relatively middling in terms of their valence (Table 1).

**Table 1:** Self-reports of elicited emotion

Stimulus category\response	Disgust	Fear	Amusement	Sexual Desire	Neutral
Disgust	**0.74**	0.10	0.10	0.00	0.06
Fear	0.02	**0.64**	0.09	0.00	0.15
Amusement	0.01	0.03	**0.78**	0.00	0.10
Sexual Desire	0.11	0.00	0.03	**0.66**	0.14
Neutral	0.01	0.02	0.05	0.00	**0.76**

Participants could select whether the videos elicited any of the emotions (Disgust, Fear, Amusement, Sexaul Desire) or none at all. Participants were allowed to choose more than one emotion

### Are the Five Emotion Categories Associated with Stable Facial Patterns?

#### Classification Performance

**Fig. 1:**
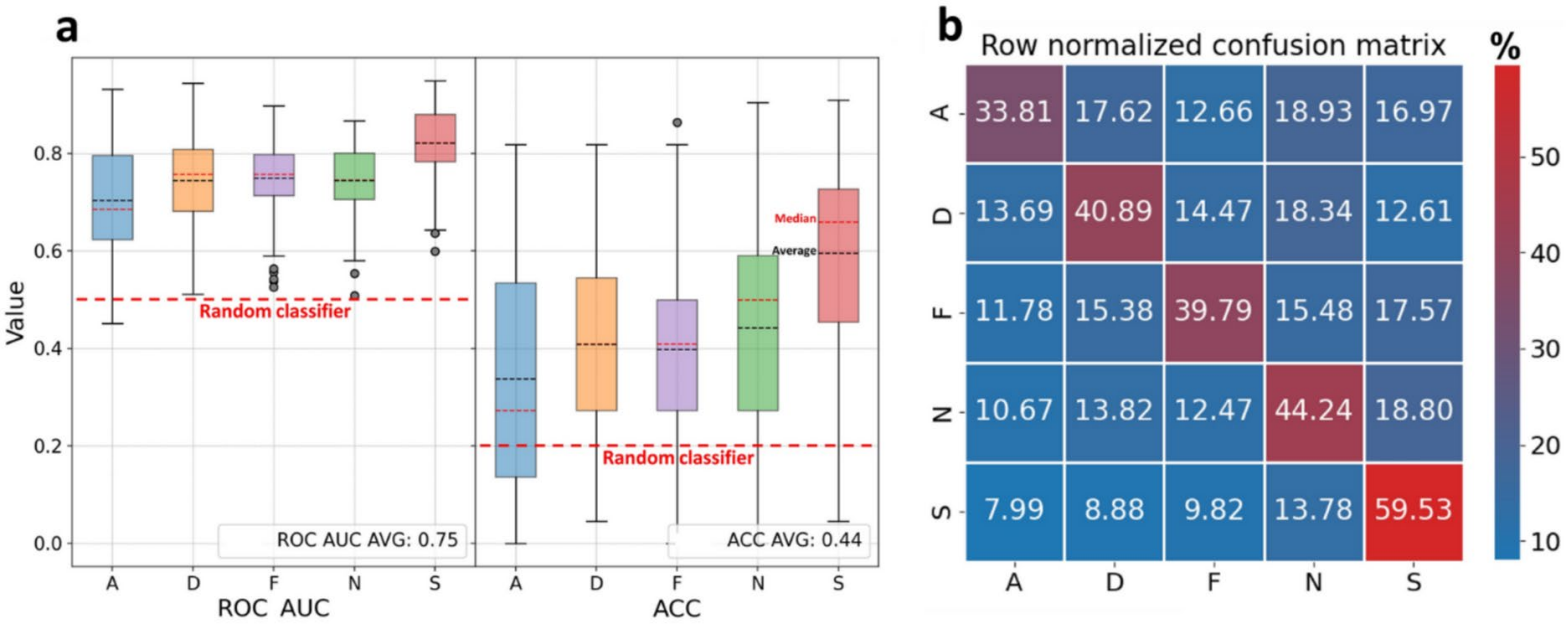
**a** The model’s statistical assessments used receiver operating characteristic area under the curve (ROC AUC) and subset accuracy (ACC) per emotion class: amusement (A), disgust (D), fear (F), sexual arousal (S), and neutral (N) as the baseline. The colored boxplot rectangles represent the interquartile range (IQR), which is equal to the difference between the upper and lower quartiles (Chattamvelli et al., 2015). Values outside the 1.5 IQR range are marked with grey dots. The red and black dashed lines inside the IQR rectangles denote the median and average, respectively. The bold red dashed line labeled “Random classifier” marks the value of an unskilled classifier, similar to a coin toss. **b** The row-normalized confusion matrix displays the median of all LOOCV confusion matrix results, which were then row-normalized (the sum of the values of each row is 100%). Note: The current figure is taken from Shvimmer et al. (2022) to present the classification results

The development of the classifier and its evaluation are reported in full in the methodological paper describing the method by Shvimmer et al. (2022). The classification results (Fig. 1; taken from Shvimmer et al. (2022)) show an out-of-all correct identification rate of 43.65% on average (vs. baseline of 20%); in particular: Neutral 44.24%, Disgust 40.89%, Fear 39.79%, Amusement 33.81%, and Sexual Desire 59.53%.

#### Human Classification Performance

Past research has questioned whether humans can reliably identify emotions from the face (Crivelli et al., 2017; Gendron et al., 2014; Russell, 2003). To see whether such results replicate in the current dataset, we examined the classification accuracies of human judges who viewed the videos of participants.

A total of 10 psychology M.A, MsC, and Ph.D students who are personnel in the Principle Investigator’s lab evaluated a randomly selected sample of the face videos and had to indicate which of the five categories they belonged to. Each judge saw a total of 100 videos, randomly selected from 20 participants, with a total of 20 videos per category. The participants were not informed about the purpose of the study and were not directly involved in the current research.

**Fig. 2:**
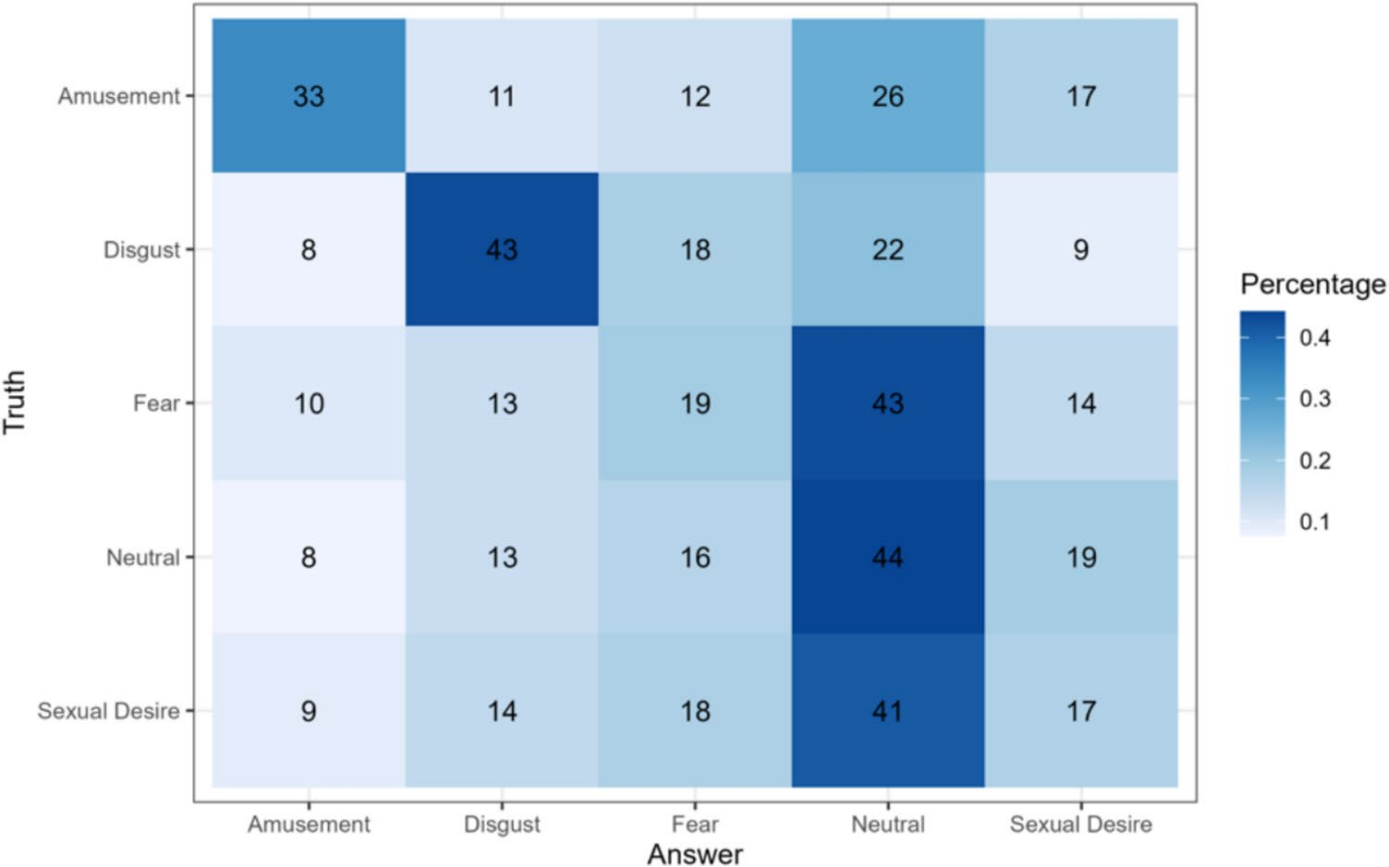
Confusion matrix depicting the classification performance of human judges in identifying emotions from face videos. The matrix shows the percentage of responses for each true emotion category (rows) classified into each predicted emotion category (columns). The diagonal elements represent the accuracy rates for each emotion category

Consistent with prior literature, judges recognized Fear (19.07%) and Sexual Desire (17.33%) at a level that is no better than chance (20%). This result markedly differs from the classifier output which was able to identify Fear and (most notably) Sexual Desire at rates higher than chance. For the remaining three categories, the performance of human judges was very similar to that of the machine learning classifier: human judges identified Neutral videos as such on 44.26 of the trials, Disgust on 44.3% of the trials, and amusement on 33.29% of the trials. The confusion matrix summarizing these results is provided in Fig. 2.

Thus, the results show that some emotions can be inferred from facial expressions (e.g., smiling for amusement), but that other emotions (sexual arousal, fear), there are no overt facial expression that people easily identify; nonetheless, despite the fact that it is not recognizable by people, there is physiological information in the human face that can be used to identify people’s emotions.

### Is the Relation Between Face Recordings and Emotion Categories Stable Across Different Stimuli?

**Fig. 3:**
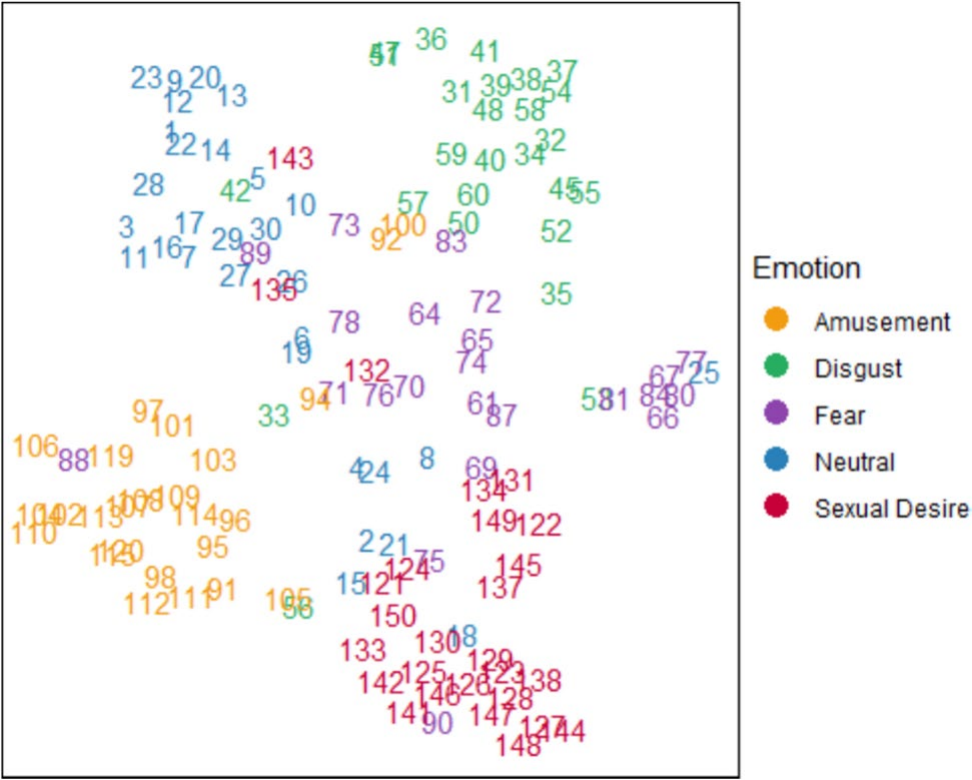
Each data point represents a video and is marked by the video index. The color of the points is determined by the validated category of the video. The t-SNE is mapped by our classification data

We projected the classification output, which contains five dimensions, to a two-dimensional space using t-SNE (Van der Maaten & Hinton, 2008; Fig. 3).

As seen in Fig. 3, the outputs of our classifier indeed seemed to generate clusters of discrete emotion labels that are relatively stable across the different stimuli. We sought to quantify the extent to which the classification outputs form clusters corresponding to true emotion labels. We followed standard practice by reporting NMI for a range of solutions (e.g., 2 to 10 clusters). After normalizing (z-scoring) the five features, we applied hierarchical clustering (*k* = 5 with complete linkage) and measured similarity using normalized mutual information (NMI), ranging from 0 (no mutual information) to 1 (exact distribution). The five-cluster solution showed a reasonable fit, with $NM{I}_{5\mathrm{D}}$ = 0.42.

The two-cluster solution ($NM{I}_{2\mathrm{D} }=$ 0.08) is significantly lower than the five-cluster solution ($NM{I}_{5\mathrm{D}}$ = 0.423), supporting the discrete emotion approach. The seven-cluster solution was slightly higher ($NM{I}_{7\mathrm{D}}$ = 0.4533) compared to the five-cluster solution (0.4234) with a *p*-value of 0.0483. Despite this statistically significant improvement, the small difference of 0.03 suggests that the seven-cluster solution does not provide substantial theoretical advancement over the five-cluster solution. Finally, as shown in Table 2, only three clusters—neutral, sexual desire, and disgust—were identified, with fear and amusement not forming distinct clusters in this analysis (however, note that such clusters were more noticebale). Importantly, while these data do not strongly support the five-dimensional solution, it is clearly preferred over the two-cluster solution as it provides a better overall fit.

**Table 2:** Hierarchical clustering (*k* = 5) of classifier outputs

Ground truth	Cluster 1	Cluster 2	Cluster 3	Cluster 4	Cluster 5
Neutral	22	7	1	0	0
Sexual Desire	3	24	0	0	0
Fear	2	5	6	9	1
Disgust	2	0	2	22	0
Amusement	5	0	0	10	10

### Can the Stability of the Relation Between Face Recordings and Emotion Categories Rely on Two Underlying Dimensions?

The previous analysis showed that the output of the classifier indeed generated five clusters that fit the true emotion label. Nonetheless, it could be argued that these five clusters emerge from a combination of two latent dimensions of core affect. One way to examine this possibility is by reducing the five-dimensional output of the face classifier to two dimensions. In light of this, we ran dimensionality reduction using PCA. If the five emotion labels can be represented on two dimensions, as suggested by the core affect model, we would be able to get similar clustering on the reduced dimensionality as we did with the full five dimensional space. The results, as shown in Table 3, showed that two principle componenets account for 72.5% of the variance in the five-dimensional space.

**Table 3:** Principle component analysis loadings

Variable	PC1	PC2	Complexity
Amusement	0.77		1.13
Digust	0.80		1.10
Fear		0.89	1.02
Neutral		− 0.80	1.06
Sexual Desire	− 0.91		1.10

**Fig. 4:**
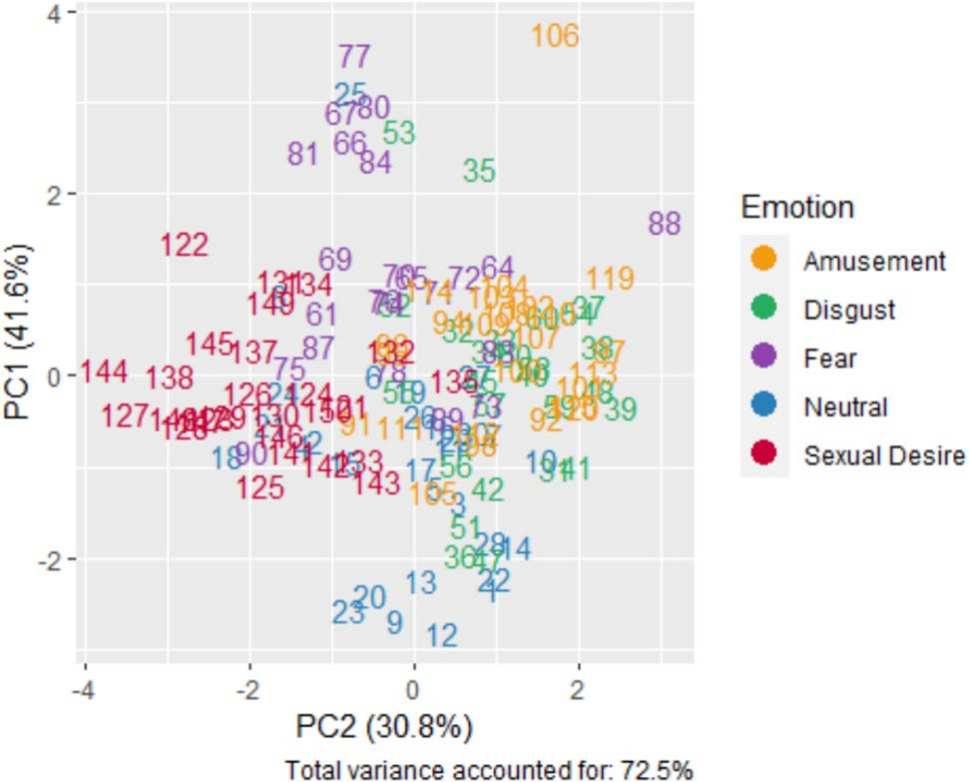
Each data point represents a video and is marked by the video index. The color of the points is determined by the validated category of the video

We re-ran the hierarchical clustering algorithm on the two-dimensional space (shown in Fig. 4). The results showed that the five-cluster solution had a reduced fit with the true Emotion label, $NM{I}_{2\mathrm{D}}=0.26$—a marked reduction of 39.0% compared to the clustering based on the five-dimensional space. A permutation test showed that the difference in fit between the two models was significant (${p}_{\mathrm{permutation}}<.003$). Thus, this analysis does not support the idea that the five clusters observed in Fig. 3 emerge from a combination of two latent dimensions of core affect.

### Are Valence and Arousal Sufficient to Explain Self-Reported Discrete Emotion Labels?

Past research has shown that the “core affect” dimensions suffice to explain much of the variance in discrete emotion labels (i.e., the circumplex model; Russell, 1980). In light of this, before turning to examine whether valence and arousal suffice to explain the classification results, we sought to examine whether they suffice to explain participants’ *self-report* data.

Since the classification output is dependent, a separate linear regression analysis was constructed for each emotion, and the ${R}^{2}$ score was calculated for all five regressions together by summing the sum square residuals and sum square total of all the regressions. We found an ${R}^{2}$ of 50.66% (Table 1). This result means that a linear model can explain about one-half of the variance of the reported discrete emotional labels using the core affect self-reported dimensions.

Linear regression models are limited, since they assume a linear relationship. Thus, we applied a non-parametric support vector regression (SVR). SVR allows more flexibility by making fewer rigid assumptions regarding the nature of the relationship between the variables by applying a non-linear function (i.e., kernel). The model’s hyperparameters were tuned using a random search. Models were evaluated using the validation set approach and 10-fold cross-validation (James et al., 2013). As seen in Table 4, the radial kernel gave the best result, with an impressive cross-validated ${R}^{2}$ of 83.14%. Thus, when it comes to explaining *self-reported* discrete emotion labels, it seems that self-reported valence and arousal suffice to explain the lion’s share of the variance in our vector of self-reported discrete emotion ratings.

**Table 4:** Linear and support vector regression—self reported emotional labels

Model type	Cross-validated ${R}^{2}$
Train LM	.535
Test LM	.506
Train SVR radial basis	.809
**Test SVR radial basis**	.831
Train SVR linear kernel	.345
Test SVR linear kernel	.338
Train SVR polynomial kernel	.508
Test SVR polynomial kernel	.458
Train SVR sigmoid kernel	.289
Test SVR sigmoid kernel	.289

The table presents the cross-validated ${R}^{2}$ score of each of our models. All models were trained and predicted on the same train and test sets to avoid biases caused by different data sets

### Can Valence and Arousal Explain Facial Patterns that Correspond to the Five Emotions Based on Our Classification?

As described in the previous analysis, participants’ self-reported valence and arousal can accurately predict self-reported discrete emotion labels. In light of this, it is crucial to examine whether self-reported valence and arousal can also accurately predict the outputs of the physiology-based classifier. If it is the case that a linear (or non-linear) combination of valence and arousal can predict the outcome, then it provides further support for the core affect view.

For that purpose, we examined whether a linear combination of valence and arousal can predict the vector of values that are the output of the physiology-based discrete emotion label classifier. First, we conducted a linear regression analysis. We found a cross-validated ${R}^{2}$ of only 9.39%. Next, an SVR model was trained using 10-folds with different kernels. The radial kernel yielded the best result, with an unimpressive cross-validated ${R}^{2}$ of 13.69% (Table 5). The cross-validated ${R}^{2}$ of both the test and train is higher in the SVR model compared to the linear model (Table 4). Meaning, 13.69% of the classifier output can be explained by the core affect model (which includes the participant’s valence and arousal self-reports).

**Table 5:** Linear and support vector regression—classifier emotional label output

Model type	Core affect model cross-validated ${R}^{2}$	Discrete model cross-validated ${R}^{2}$	Core affect and discrete model combined cross-validated ${R}^{2}$
Train LM	.167	.487	.459
**Test LM**	.093	**.422**	.412
Train SVR radial basis	.213	.485	.497
**Test SVR radial basis**	**.136**	.415	.415
Train SVR linear kernel	.134	.466	.468
Test SVR linear kernel	.062	.409	.406
Train SVR polynomial kernel	.027	.398	.427
Test SVR polynomial kernel	− .036	.324	.309
Train SVR sigmoid kernel	.134	.466	.468
Test SVR sigmoid kernel	.062	.409	.406

The table presents the cross-validated ${R}^{2}$ of each of our models. All models were trained and predicted on the same train and test sets to avoid biases caused by different data sets

After we examined whether valence and arousal can explain our classification of emotional experience, we wanted to examine if we can explain our classification better by the discrete emotion self-reports. For that purpose, we conducted the same analysis as with the core affect approach. We compared all the models we used in the previous section (Table 5) and found that the cross-validated ${R}^{2}$ score of both the test and train is higher in the discrete model compared to the core affect model (Table 5). The discrete model explains 42.27% of the variance, namely, there is a non-negligible 28.58% of the variance that is not captured by valence and arousal. In other words, “core affect” does not suffice to explain the ability of a purely physiology-based classifier to infer discrete emotional labels.

**Fig. 5:**
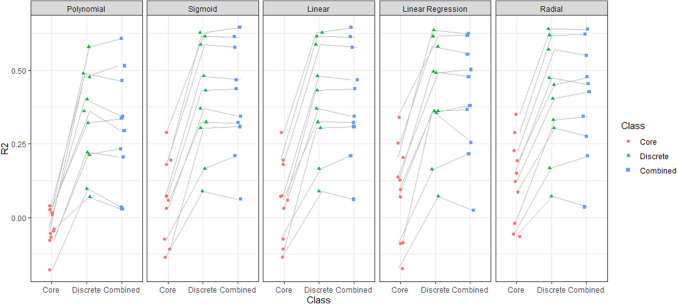
Each data point represents an ${R}^{2}$ of onefold in a specific model, while each grey line connects the ${R}^{2}$ values from the same fold. The color of the points is determined by the model type (core affect; discrete; combined core affect and discrete)

Finally, in order to estimate whether the difference between the prediction accuracy of combined core affect and discrete model is significant, we calculated the ${R}^{2}$ for each fold in each of our models (Fig. 5) to examine the level of overlap between the ${R}^{2}$ values. This analysis demonstrated that the discrete model was superior to the core affect model.

Finally, it could be argued that our valence and arousal measurements for the videos are noisier than the emotional labels rating. If that is the case, then the lower explanatory value of valence and arousal ratings could simply be a result of inaccurate measurement of these constructs. Thus, we sought to calculate the reliability of our valence and arousal estimates. Given that arousal and valence ratings were based on an aggregation of 110 participants, we conducted 10 bootstrapping runs wherein we randomly divided our sample into two sub-samples, calculated the mean valence and arousal for each video for the two sub-samples, and then calculated the correlations between the two sub-samples. The results showed that for both valence and arousal, the average correlation was 0.95. Namely, our stimulus level estimates of valence of arousal were highly reliable, and nonetheless, these dimensions do not suffice to explain the ability of the classifier to identify the emotions participants experienced.

## Discussion

In the current study, we examined whether there are objectively measured patterns in the face that can predict discrete emotional labels that are not reducible to core affect (i.e., valence and arousal). Recent research (Shvimmer et al., 2022) described a method whereby emotion labels can be classified from the face with good accuracy based on biological information gleaned from the face. However, a major limitation of this past work is that it remained possible that the accurate classification of emotion labels is solely explained by valence and arousal, as suggested by the “core affect” view. By re-analyzing the results of Shvimmer et al. (2022), the current findings provide strong evidence against this possibility and, thereby, are more in line with a “maximalist view,” according to which bodily information can indeed be used to predict people’s emotional labels.

Consistent with the circumplex model (Russell, 1980), we found that valence and arousal suffice to explain more than 80% of the variance in participants’ self-reports of discrete emotion labels. This finding is in line with research that found valence and arousal as key dimensions that lie at the core of affective experience (Barrett et al., 1998; Bradley et al., 1992; Russell & Barrett, 1999). However, importantly, when looking at the outputs of the emotion classifier (that rely on objective, bodily patterns) —valence and arousal accounted for 13% of the variance, whereas discrete emotion ratings accounted for 42% of the variance of our emotion classifier output. Thus, there are stable, reliable, biological patterns that are predictive of our discrete emotion labels that are *not* reducible to reported core affect.

Our findings challenge previous research, suggesting that facial information primarily conveys affective properties such as valence and arousal, rather than discrete emotional states (e.g., Barrett et al., 2019; Crivelli et al., 2017; Gendron et al., 2014; Russell, 2003). While these studies focus on perceptible changes in facial expressions and their role in social communication, our results indicate that both perceptible and non-perceptible facial signals can provide information about discrete emotional experiences. Specifically, our findings align with research demonstrating that bodily signals, including subtle and non-perceptible changes, can predict discrete emotional experience labels (e.g., Cowen & Keltner, 2017; Horikawa et al., 2020; Sauter, 2017). This suggests that even when facial changes are not overtly perceptible, they may still play a role in conveying discrete emotional information, thus contributing to the broader debate on the physiological correlates of emotions. The idea that emotions are associated with specific facial patterns dates back to Darwin (1872), who claimed that some emotions have universal facial expressions and proposed principles explaining why particular expressions occur for particular emotions. This claim was supported by Ekman (1984) and Izard (2007), who argued for universality in facial expressions of basic emotions. However, it should be noted that the fact that the five emotions produced facial patterns that could be distinguished from each other does not necessarily mean that our results are inconsistent with dimensional theories (Mehrabian, 1980; Scherer, 2009). All that is shown in the present research is that facial patterns reliably associated with different discrete emotion labels cannot be readily explained with a simple two-dimensional model of core affect. In other words, while the results are more consistent with a maximalist view, they do provide support to either the basic emotion view or dimensional theories.

Because facial expressions are subserved by physiological changes (i.e., muscle contractions that are associated with changes in blood flow), by gauging physiological changes, we measured both overt expression, minute muscle contractions (i.e., so-called micro-expressions), as well as physiological changes that do not involve any muscle contraction. Due to this inherent confound between facial expression and muscle contortion\changes in blood flow and content, it is difficult to definitely gauge the extent to which the classifier relied on relatively overt expressions vs. relatively covert physiological signals. However, the results of human classification suggest that while in some cases (e.g., Amusement and Disgust) overt expressions were visible and have likely served as an important basis for the classifier, in other emotions (Fear and Sexual Desire), there were no readily available expressions, and the classifier likely succeeded by relying on other types of information.

While the results show that there is information in the human face that could be used to classify people’s discrete emotional labels, the results do not tell us whether this information stems from processes that are causal to the emotion generation process or are epiphenomenal hints. By analogy, a person who is nervous, may speak quickly, but this behavior—while potentially hinting about his mental state to a certain extent—is not constitutive nor essential in the experience of nervousness.

## Constraints on Generality

A limitation of the current study is that we used a relatively small set of emotions, containing five discrete emotions and two affective dimensions. Recent research suggests that people may construe at least 27 distinct categories of emotions that capture an increasingly complex array of states associated with distinct experiences (Cowen & Keltner, 2017). This limitation should be addressed in future research by selecting a bigger set of emotional states and affective dimensions.

The conclusions from the study directly pertain to the specific situation wherein the emotional induction is somewhat lengthy (i.e., in the current study, blocks of five emotion-eliciting stimuli, generating blocks of about half a minute wherein a given emotional state is induced). This result may not generalize to situations wherein emotions are induced by single short-lived events (e.g., a surprising “jump scare” in horror movies, which may yield more classic fear expressions).

Another limitation of the study pertains to the demographics of the sample. The study was conducted using a large sample of participants in a specialized laboratory located at Ben Gurion University. As a result, the participants were drawn from a random sample of psychology students, which may restrict the generalizability of our findings to broader populations. Furthermore, it is important to note that all participants in the study were Israeli and fell within the age range of 18–33. To extend the applicability of our results, future research should aim to replicate our findings across different countries and age groups.

## Conclusion

In conclusion, the current result show that there is information in the face that can be used to classify self-reported discrete emotion label and that this information is not reducible to valence and arousal; as such, our findings are inconsistent with the “core affect view.” While such findings do not provide direct support for the discrete\basic emotional view, the current piece of evidence contributes to the debate concerning the nature of emotional experience.

## Additional Information

### Data Availability

The datasets generated and analyzed during the current study are not publicly available because it contains sensitive information about the subjects. Part of the data will be made available from the corresponding author upon reasonable request.
